# Ischemic Stroke at a Tertiary Academic Hospital in Tanzania: A Prospective Cohort Study With a Focus on Presumed Large Vessel Occlusion

**DOI:** 10.3389/fneur.2022.882928

**Published:** 2022-07-14

**Authors:** Sarah Shali Matuja, Rashid Ali Ahmed, Patricia Munseri, Khuzeima Khanbhai, Kezia Tessua, Frederick Lyimo, Gustavo J. Rodriguez, Vikas Gupta, Alberto Maud, Mohammad Rauf Chaudhury, Mohamed Manji, Faheem Sheriff

**Affiliations:** ^1^Department of Internal Medicine, Catholic University of Health and Allied Sciences, Mwanza, Tanzania; ^2^Department of Neurology, Massachusetts General Hospital and Harvard Medical School, Boston, MA, United States; ^3^Department of Internal Medicine, Muhimbili University of Health and Allied Sciences, Dar es Salaam, Tanzania; ^4^Department of Cardiology, Jakaya Kikwete Cardiac Institute, Dar es Salaam, Tanzania; ^5^Department of Internal Medicine, Ocean Road Cancer Institute, Dar es Salaam, Tanzania; ^6^Department of Radiology, Muhimbili National Hospital, Dar es Salaam, Tanzania; ^7^Department of Neurology, Texas Tech University Health Sciences Center, Paul L Foster School of Medicine El Paso, El Paso, TX, United States

**Keywords:** ischemic stroke, large vessel occlusion, thrombectomy, morbidity and mortality, Tanzania

## Abstract

**Background:**

Large vessel ischemic strokes account for more than one-third of all strokes associated with substantial morbidity and mortality without early intervention. The incidence of large vessel occlusion (LVO) is not known in sub-Saharan Africa (SSA). Definitive vessel imaging is not routinely available in resource-limited settings.

**Aims:**

We aimed to investigate the burden and outcomes of presumed LVO among patients with ischemic stroke admitted to a large tertiary academic hospital in Tanzania.

**Methods:**

This cohort study recruited all consenting first-ever ischemic stroke participants admitted at a tertiary hospital in Tanzania. Demographic data were recorded, and participants were followed up to 1 year using the modified Rankin Scale (mRS). A diagnosis of presumed LVO was made by a diagnostic neuroradiologist and interventional neurologist based on contiguous ischemic changes in a pattern consistent with proximal LVO on a non-contrast computed tomography head. We examined factors associated with presumed LVO using logistic regression analysis. Inter-observer Kappa was calculated.

**Results:**

We enrolled 158 first-ever ischemic strokes over 8 months with a mean age of 59.7 years. Presumed LVO accounted for 39.2% [95% confidence interval (CI) 31.6–47.3%] and an overall meantime from the onset of stroke symptoms to hospital arrival was 1.74 days. Participants with presumed LVO were more likely to involve the middle cerebral artery (MCA) territory (70.9%), *p* < 0.0001. Independent factors on multivariate analysis associated with presumed LVO were hypertension [adjusted odds ratio (aOR) 5.*74* (95% CI: 1.74–18.9)] and increased waist-hip ratio [aOR 7.20 (95% CI: 1.83–28.2)]. One-year mortality in presumed LVO was 80% when compared with 73.1% in participants without presumed LVO. The Cohen's Kappa inter-observer reliability between the diagnostic neuroradiologist and interventional neurologist was 0.847.

**Conclusion:**

There is a high burden of presumed LVO associated with high rates of 1-year morbidity and mortality at a tertiary academic hospital in Tanzania. Efforts are needed to confirm these findings with definitive vessel imaging, promoting cost-effective preventive strategies to reduce the burden of non-communicable diseases (NCDs), and a call for adopting endovascular therapies to reduce morbidity and mortality.

## Introduction

Stroke is a leading cause of death and disability, particularly in low and middle-income countries (LMICs), contributing to 80% of all incident cases, 87% of all deaths, and 89% of stroke-related disability-adjusted life years (DALYs) (1, 2). According to the 2019 Global Burden of Disease (GBD) report, there were 12.2 million stroke incident cases, 6.55 million stroke fatal cases, and 143 million stroke DALYs (3). Globally, the proportions of ischemic strokes (62.4%) are higher as compared to intracerebral hemorrhages (27.9%) and subarachnoid hemorrhages (9.7%) (3). In LMIC, ischemic stroke accounts for approximately 7 million cases (63%) and 1.5 (57%) million deaths as a proportion of all strokes and deaths, respectively (4). A similar trend is observed in sub-Saharan Africa (SSA), where ischemic strokes account for 68% of all strokes as compared to 32% of hemorrhagic strokes (5).

Large vessel occlusions (LVOs) account for 20–40% of all ischemic strokes associated with substantial morbidity and mortality (6, 7). In high-income countries (HICs), the incidence of LVO is estimated at 24 per 100,000-person years, summing up to 80,000 cases annually (8). In the pre-endovascular era, mortality was two times (64% vs. 24%) among those with LVO as compared to those without, respectively (6). This eventually led to the adoption of endovascular interventions that have proven cost-effective in preventing mortality and disability from a stroke in HIC based on previous pivotal clinical trials (9, 10). Notably, such resources are limited in the vast majority of facilities sub-serving people living in SSA contributing to increased stroke mortality. In addition, it is unusual for patients to arrive in the early time window for stroke intervention. Little is known about the true burden and outcomes of LVO among patients with ischemic stroke in SSA. We therefore aimed to investigate the prevalence and outcomes of first-ever ischemic strokes, with a particular focus on presumed LVO in patients admitted to a tertiary academic hospital in Tanzania.

## Materials and Methods

### Study Design and Population

This cohort study was conducted at Muhimbili University of Health and Allied Sciences Academic Medical Center (MAMC), medical wards in Dar es Salaam, Tanzania. This is a tertiary academic hospital that receives referral patients from both public and private hospitals and offers specialized medical care for all medical subspecialties in Tanzania.

Consecutive participants aged ≥18 years admitted at MAMC with a diagnosis of first-ever ischemic stroke according to the World Health Organization definition (WHO) (11) between June 2018 and January 2019 were recruited. Written informed consent was obtained from either the participants or their next of kin if the participant was unable to consent before study enrollment.

### Data Collection

An interviewer-based structured questionnaire was administered to all study participants or their caregivers capturing the following: demographic information, date of onset of stroke symptoms, date of admission, contact details, and premorbid stroke risk factors (e.g., hypertension, diabetes mellitus (DM), and HIV infection). Medication history for hypertension, DM, HIV, and hormonal contraception for women was also obtained. We also inquired about smoking and alcohol consumption.

### Clinical Measurements

Physical examination included measurement of blood pressure (BP) using a standard digital BP machine, AD Medical Inc. Three BP readings were collected spaced 5-min apart, while the participant was at rest, and an average BP was computed. Participants were regarded as hypertension when the average BP readings for systolic blood pressure (SBP) ≥140 mmHg or diastolic blood pressure (DBP) ≥90 mmHg or if the participant was on anti-hypertensive therapy according to the Joint National Committee 7 (JNC-7) definition (12). All participants had their waist and hip circumference measured using a tape measure and recorded in centimeters. The waist-hip ratio was interpreted according to the WHO guidelines; in men, the ratio of ≥0.90 and in women, the ratio of ≥0.85 were regarded as substantially increased (13). The examination also included precordial and neck carotid auscultation.

### Laboratory Investigations

Capillary fingertip blood samples were collected to check for random blood glucose (RBG) levels and HIV rapid testing using a glucometer GLUCOPLUS^TM^ and SD Bioline, respectively. A fasting blood glucose (FBG) sample was collected the following morning for participants with RBG levels of ≥11.1 mmol/l (equivalent to ≥200 mg/dl). DM diagnosis was defined as an RBG reading of ≥11.1 mmol/l (equivalent to ≥200 mg/dl) or an FBG reading of ≥7 mmol/l (equivalent to ≥126 mg/dl). HIV testing was performed using sequential rapid test SD Bioline, followed by Unigold Biotech – these are both rapid immune-chromatographic antibody assays for HIV 1/2 antibodies.

We aseptically collected 5 ml of venous blood from each study participant. We analyzed random total cholesterol, triglycerides (TGA), low-density lipoproteins (LDL), and high-density lipoproteins (HDL) using machine model A15 of BioSystems. Cutoffs for total cholesterol >240 mg/dl were regarded as hypercholesterolemia, TGA >200 mg/dl as hypertriglyceridemia, LDL >129 mg/dl as increased, and HDL <35 mg/dl as reduced levels.

### Brain Imaging

**Figure 1:**
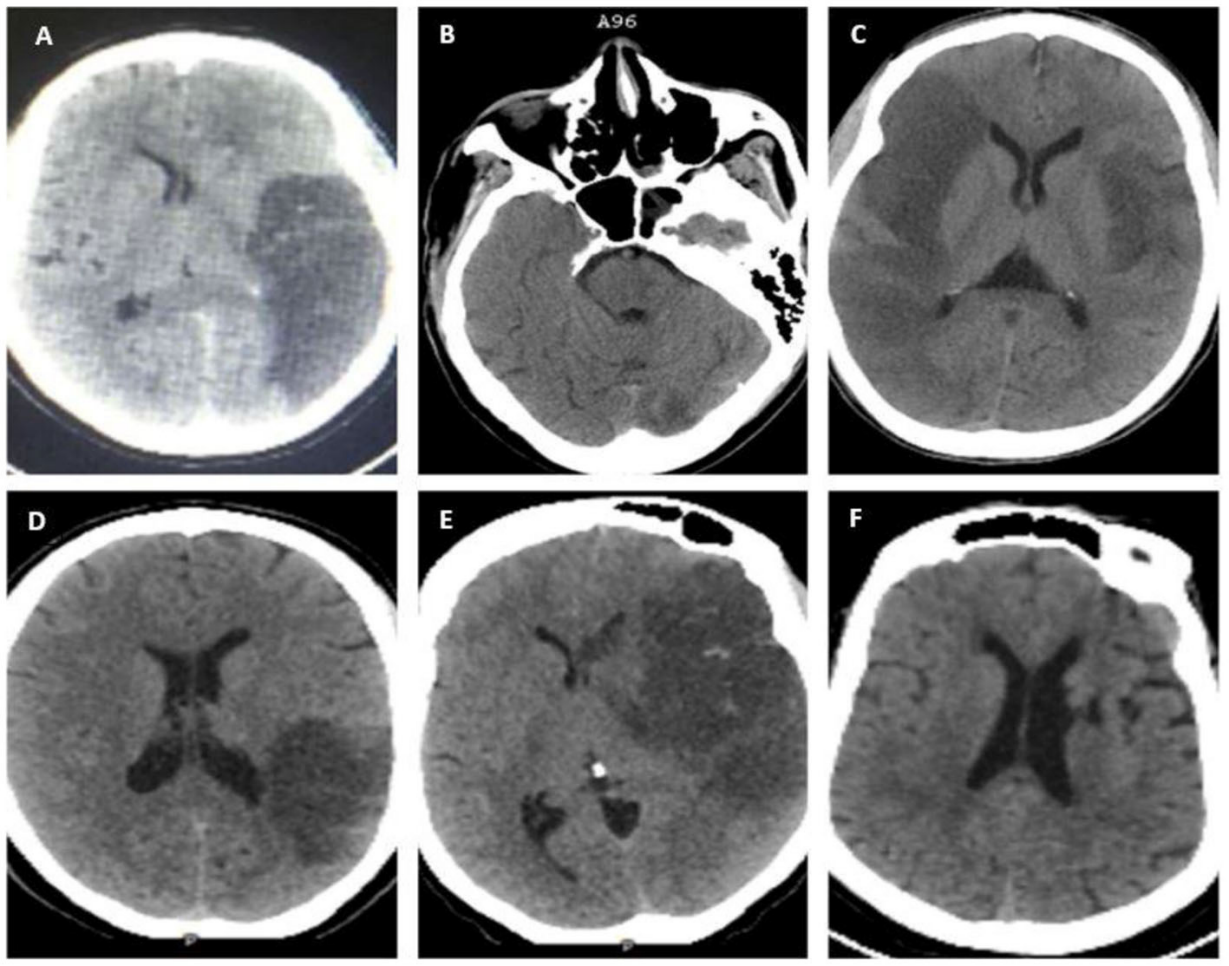
Non-contrast brain computed tomography (NCCT) scans showing evidence of ischemic infarcts in various vascular territories; **(A)** left distal middle cerebral artery (MCA) M1 with HI 1; **(B)** basilar and left posterior cerebral artery (PCA) infarction with a hyperdense basilar sign; **(C)** bilateral MCA: right M1 and left distal M1/ proximal M2; **(D)** inferior division M2 MCA with HI 1; **(E)** left proximal M1 with HI 1; and **(F)** left Lacunar infarct (non-LVO).

A non-contrast brain computed tomography (NCCT) using GE Healthcare Optima was performed on all the study participants only at baseline, and images were independently interpreted by a diagnostic neuroradiologist (FL) and an interventional neurologist (FS). Ischemic stroke was defined based on a clinical stroke syndrome and objective evidence of focal cerebral ischemia in a vascular territory by an NCCT (14) (Figure 1). Presumed LVO was defined as occlusion of the proximal segments of the MCA (i.e., M1 or proximal M2), anterior cerebral arteries, posterior cerebral arteries, vertebral arteries, and basilar arteries that were determined radiographically based on contiguous hypo-density noted on NCCT that can be attributed to the involved vascular territory with or without the presence of additional findings, such as hyperdense MCA or basilar signs (15, 16). Hemorrhagic transformation was defined as per European Cooperative Acute Stroke Study (ECASS II) (17). Hemorrhagic infarction type 1 (HI1) was defined as petechial hemorrhages at the infarct margins. Hemorrhagic infarction type 2 (HI2) was defined as petechial hemorrhages throughout the infarct and no mass-effect was attributable to the hemorrhages. Parenchymal hematoma type 1 (PH1) was defined as ≤ 30% of the infarcted area and minor mass effect attributable to the hematoma. Parenchymal hematoma type 2 (PH2) was defined as >30% of the infarct zone and substantial mass effect attributable to the hematoma. Midline shift was defined as any measurable shift of midline cerebral structures seen on axial images of an NCCT, specifically the septum pellucidum and/or the pineal gland (18).

### Cardiovascular Assessment

Transthoracic echocardiography (ECHO) using GE Medical Systems was performed by a trained cardiologist, and interpretation was based on European Society of Cardiology/American Society of Echocardiography guidelines for evidence of any structural heart abnormalities and other cardiac risk factors for stroke, such as mitral stenosis, presence of vegetations and thrombus (19). A 12-lead electrocardiogram (ECG) using a machine of Bionet was performed on the study participants to look for evidence of atrial fibrillation.

### Stroke Outcomes

Stroke severity was assessed using the National Institute of Health Stroke Scale (NIHSS) on admission (11). Stroke outcomes were categorized using the modified Rankin Scale (mRS) (11) at 24 h, 30 days, and 1 year from admission, with scores ranging from 0 (no symptoms) to 6 (death).

### Study Variables

The dependent variable was presumed LVO. The independent variables included demographic characteristics, risk factors (hypertension, DM, smoking, cardiac disease, alcohol consumption, increased waist-hip ratio, hypercholesterolemia, and increased LDL), vascular territories, and stroke outcomes (death or survival with/without disabilities).

### Data Analysis

Data were analyzed using SPSS version 20.0. Continuous variables were summarized and presented as means and standard deviation (SD) or medians with interquartile range [IQR]. Categorical variables were summarized as frequencies and proportions. Comparisons between proportions were done using Pearson's Chi-square test or Fisher's exact test. The logistic regression technique was used to determine independent factors associated with presumed LVO. All covariates with a *p*-value of <0.2 in the bivariable analysis were included in the multivariable analysis model. Unadjusted and adjusted odds ratios (OR), 95% confidence intervals (CI), and corresponding *p*-values were obtained from the models. A two-tailed significance level was set as a *p*-value of ≤ 0.05. Inter-observer Kappa between the diagnostic neuroradiologist (FL) and interventional neurologist (FS) was also calculated.

## Results

**Figure 2:**
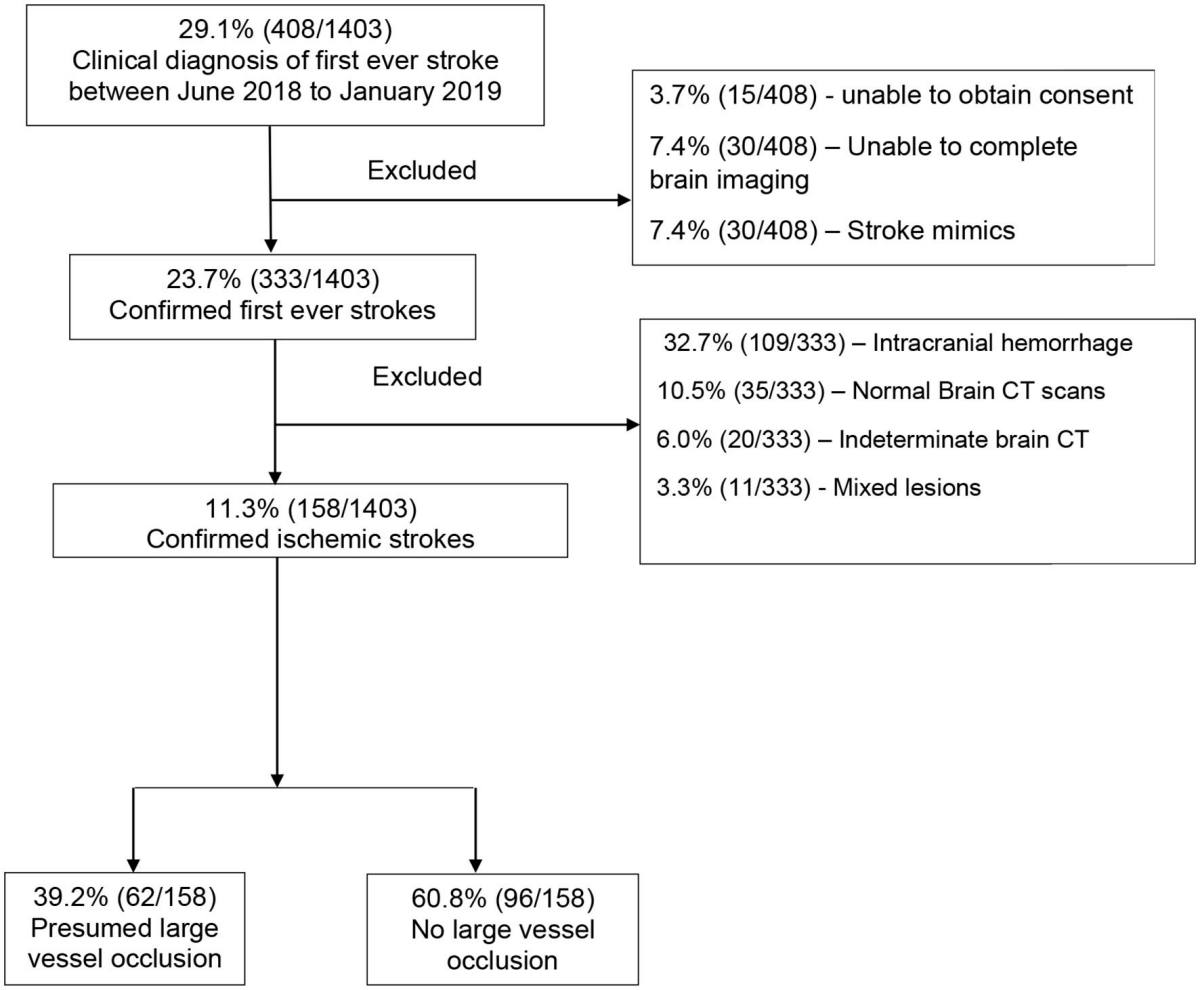
Consort diagram showing the flow of participants.

Between June 2018 to January 2019, there were 1,403 medical admissions, out of which 408 (29.1%) participants met the WHO clinical definition of first-ever ischemic stroke. We excluded 250 (15.9%) participants for the following reasons: inability to consent, those who did not complete brain imaging, those with stroke mimics based on brain imaging, intracranial hemorrhage, normal brain CT scans, indeterminate CT scans (those with artifacts or poor imaging quality), and those with mixed lesions. We recruited the remaining 158 (38.7%) participants with a confirmed diagnosis of ischemic stroke, as shown in Figure 2. The proportion of ischemic strokes over total admissions was 158 of 1,403 [(11.3%) (95% CI 9.7–13.0%)]. Of these, 62 of 158 [(39.2%) (95% CI 31.6–47.3%)] had presumed LVO.

The overall mean age ± SD of the recruited participants was 59.7 ± 16.6 years, and the majority resided in Dar-es-Salaam (72.8%), a former capital city. A quarter possessed health insurance (25.9%). The overall meantime from the onset of stroke symptoms to hospital arrival was 1.74 days with no statistically significant difference in the meantime from the onset of stroke symptoms to hospital arrival in the presumed LVO vs. non-LVO groups (1.87 days vs. 1.83, respectively, *p* < 0.32.). Overall, 29 (18.3%) participants were observed to arrive at the hospital within 24 h from stroke symptom onset with no participant arriving in <4.5-h and <8-h windows.

The MCA was the predominant major vascular territory involved. It was statistically seen more frequently in the presumed LVO group as compared to those with non-LVO, i.e., 44 (71.0%) vs. 32 (33.3%), *p* < 0.0001, respectively. Similarly, participants with presumed LVO were statistically more likely to have the following cardiovascular risk factors as compared to those with non-LVO: increased waist-hip ratio 54 (87%) vs. 56 (58.3%), *p* < 0.001, hypertension 49 (79.0%) vs. 47 (48.9%), *p* < 0.001, mean total cholesterol of 220.1 ± 95.9 mg/dl vs. 188.9 ± 56.2 mg/dl, *p* < 0.001, and mean TGA 138.4 ± 103.2 mg/dl vs. 117.1 ± 56.9 mg/dl, *p* < 0.001, respectively (Table 1).

**Table 1:** Comparison of baseline characteristics and risk factors.

**Variable**	**Presumed LVO (*n* = 62)**	**Non-LVO (*n* = 96)**	***p*-value**
Age (mean ± SD)	62.1 ± 17.4	58.0 ± 15.9	0.44
Female	39 (40.6%)	57 (59.3%)	0.81
Admission NIHSS (mean ± SD)	21.9 ± 8.4	20.4 ± 8.7	0.50
**Vascular territory**			
MCA	44 (70.9%)	32 (33.3%)	–
ACA	2 (3.22%)	3 (3.12%)	
ACA + MCA	5 (8.06%)	0	
Basilar/PCA	11 (17.7%)	8 (8.33%)	
Vertebral/PICA	0	2 (2.08%)	
Multiple	0	6 (6.25%)	
Lenticulostriate	0	11 (11.4%)	
Other**	0	34 (35.4%)	
**Vascular territory**			
MCA	44 (71.0%)	32 (33.3%)	<0.0001*
Other vascular territories	18 (29.0%)	64 (66.0%)	
Last seen normal to hospital arrival (in days)	1.71 ± 1.68	1.80 ± 1.81	0.52
Last seen normal to picture (in days)	1.87 ± 1.80	1.83 ± 2.0	0.32
Glucose - RBG in mmol/l	8.20 ± 2.55	7.55 ± 2.41	0.63
Lipids - Total cholesterol (mg/dl)	220.1 ± 95.9	188.9 ± 56.2	<0.0001*
Low-density lipoproteins (mg/dl)	89.6 ± 55.6	74.7 ± 54.0	0.80
High-density lipoproteins (mg/dl)	48.1 ± 25.5	52.2 ± 21.6	0.19
Triglycerides (mg/dl)	138.4 ± 103.2	117.1 ± 56.9	<0.0001*
Waist-to-hip ratio Increased	54(87.0%)	56 (58.3%)	<0.0001*
Hypertension	49 (79.0%)	47 (48.9%)	<0.0001*
Diabetes	10 (16.1%)	19 (19.7%)	0.55
Atrial fibrillation	9 (16.6%)	4 (5.26%)	0.05
HIV	1 (1.61%)	7 (7.29%)	0.16
Mitral stenosis	1 (1.66%)	1 (1.04%)	0.76
Carotid bruit	2 (3.22%)	2 (2.08%)	0.67
Cigarette smoking	5 (8.1%)	7 (7.29%)	0.85
Alcohol	18 (29.0%)	16 (16.6%)	0.08
Oral contraceptives	5 (13.5%)	16 (27.5%)	0.08

*Statistically significant p-value; MCA, middle cerebral artery; ACA, anterior cerebral artery; PCA, posterior cerebral artery; PICA, posterior inferior cerebellar artery; RBG, random blood glucose. **Watershed infarcts, pontine perforator infarct, thalamoperforator infarct and bilateral thalamic infarcts.

Predictors of presumed LVO are summarized in Table 2. In univariate analysis, factors that were significantly associated with presumed LVO were medical co-morbidities, such as hypertension, atrial fibrillation, increased waist-to-hip ratio, total cholesterol, and TGA. In multivariate analysis, presumed LVO was independently associated with hypertension [adjusted OR (aOR) 5.74 (95% confidence interval (CI): 1.74–18.9)] and increased waist-hip ratio [adjusted OR 7.20 (95% CI: 1.83–28.2)].

**Table 2:** Predictors of presumed large vessel occlusion.

**Variable**	**Unadjusted OR (95% CI)**	***p*-value**	Adjusted* **OR (95% CI)**	***p*-value**
Hypertension	3.93 (1.87–8.24)	0.0004	5.74 (1.74–18.9)	0.004
Atrial fibrillation	3.60 (1.025–12.6)	0.04	2.75 (0.28–27.1)	0.38
waist-to-hip ratio	4.82 (2.04–11.4)	0.0004	7.20 (1.83–28.2)	0.005
Triglycerides	0.997 (0.992–1.001)	0.12	0.998 (0.990–1.007)	0.71
Total cholesterol	0.994 (0.990–0.999)	0.02	0.993 (0.985–1.001)	0.08

*Model adjusted with age, gender, vascular territory, and statistically significant medical comorbidities: hypertension, atrial fibrillation, waist-to-hip ratio, total cholesterol, and triglycerides.

Table 3 describes the outcomes of the study participants. At 30 days, there were 3 participants who were lost to follow-up and were excluded from the analysis. Overall, the 30-day mortality was 89/155 (57.4%), with no statistically significant difference in mortality in the presumed LVO and non-LVO groups, 36/62 (58.1%) vs. 53/93 (57%), *p* = 0.51, respectively. At 1 year, there were 5 participants who were lost to follow-up and were excluded from the analysis. The overall 1-year mortality was 116/153 (75.8%), 48/60 (80%) in the presumed LVO group vs. 68/93 (73.1%) in the non-LVO group, *p* = 0.25. Those with presumed LVO were statistically more likely to have a hemorrhagic transformation on the NCCT head as compared to those with non-LVO, 29 (46.7%) vs. 5 (5.20%), *p* < 0.001, respectively. The Cohen's Kappa inter-observer reliability between the diagnostic neuroradiologist and interventional neurologist was calculated at 0.847.

**Table 3:** A comparison of outcomes among participants with and without presumed LVO.

**Outcomes**	**Presumed LVO**	**Non-LVO**	***p*-value**
**Midline shift**	10 (6.32%)	0	–
**Hemorrhagic transformation categories**			–
Hemorrhagic infarction type 1 (HI1)	23 (37.0)	3 (3.1%)	
Hemorrhagic infarction type 2 (HI2)	5 (8.0%)	2 (2.1%)	
Parenchymal hematoma type 1 (PH1)	1 (1.61%)	0	
Parenchymal hematoma type 2 (PH2)	0	0	
**Overall Hemorrhagic Transformation**	29 (46.7%)	5 (5.20%)	<0.0001
mRS*categories at 24 h			
0–3	(1.61%)	4 (4.16%)	0.33
4–6	61 (98.3%)	92 (95.8%)	
mRS*categories at 30-days	Frequency Missing: 3		
0–3	8 (12.9%)	17 (18.3%)	0.50
4–6	54 (87.1%)	76 (81.7%)	
mRS*categories at 1-year	Frequency Missing: 5		
0–3	9 (15%)	23 (24.7%)	0.12
4–6	51 (85%)	70 (75.3%)	

*mRS, modified Rankin Scale.

## Discussion

This is the first study looking at the burden and outcomes of presumed LVO among all presenting ischemic strokes in Tanzania, an East African nation representative of many LMICs located in SSA. The present study was conducted at a tertiary specialized academic hospital and found a proportion of 39.2% of presumed LVO. This is a large proportion of all presenting ischemic strokes and is comparable to a previously reported global prevalence of 31% in a large meta-analysis (20). Our findings are of particular importance given the high burden of ischemic strokes in Tanzania (21) and the need for good epidemiological data before advocating for evidence-based acute stroke interventions in resource-limited settings. There is currently no data on the prevalence of LVO ischemic strokes in SSA.

The mechanisms for LVO include intracranial artery atherosclerosis, cardioembolism, artery to artery embolism (from extracranial atherosclerosis or dissection), and unknown/cryptogenic causes (22). Our study found that risk factors for presumed LVO on the univariate analysis included hypertension, increased waist-to-hip ratio (a surrogate for obesity), increased total cholesterol, triglycerides, and atrial fibrillation. On multivariate analysis, only hypertension and increased waist-to-hip ratio remained statistically significant, which are known risk factors for both intracranial and extracranial vessel atherosclerosis (23). It is notable that our study showed a relatively lower prevalence of atrial fibrillation of 16.6% in the presumed LVO group and 5.3% in the non-LVO group when compared to a meta-analysis of 5 randomized trials that looked at the efficacy of endovascular thrombectomy over standard medical care where the prevalence of atrial fibrillation was noted to be 33% (24). A similar high prevalence of atrial fibrillation was noted in the DAWN trial with 40% in the mechanical thrombectomy arm and 24% in the medical therapy arm (25). Several factors might explain the lower prevalence of atrial fibrillation in this setting that included genetic predisposition, under surveillance resulting in lower detection of occult or paroxysmal atrial fibrillation using conventional resting ECG that was utilized as a part of routine clinical care and poor access to healthcare (26, 27). Stroke is preventable in SSA by ensuring early detection and control of modifiable risk factors, related to urbanization and lifestyle changes. Therefore, efforts need to be centered on promoting low-cost interventions to reduce this looming epidemic of non-communicable diseases (NCDs) since a stroke in Tanzania is associated with substantial morbidity and mortality, impacting the country's economy (28). The involvement of the MCA as the most common site for presumed LVO is in concordance with global trends (29).

The mainstay for the management of LVO is through endovascular interventions, which have been associated with improved outcomes; however, the majority of these clinical trials on the efficacy of thrombectomy have been done in HIC (9, 10). The barriers in LMIC that lead to inequity in endovascular care are mainly due to the high cost of the procedure, lack of endovascular specialists, and late times to presentation, among others. This was shown in the Mexican Endovascular Reperfusion Registry (MERR), which supported the effectiveness of thrombectomy but noted that treatment was mainly feasible in private hospitals (30). Similarly, the RESILIENT trial conducted in Brazil was the first clinical trial that evaluated the benefits of thrombectomy in patients with LVO in LMIC presenting within 8 h from stroke symptoms (31). Their results showed a better functional outcome at 90 days among the LVO group receiving endovascular therapy. Muhimbili National Hospital (MNH) and MAMC, where this study was conducted, can administer IV thrombolysis to patients presenting with an acute coronary syndrome, which will potentially translate to IV thrombolysis for acute ischemic stroke; however, we currently do not have mechanical thrombectomy capability in Tanzania. The poor functional outcomes in this cohort could be attributed to the severity of the stroke. It is notable that both groups had similar NIHSS scores (mean NIHSS score >20), which is a matter of concern, especially in the non-LVO group. One possible explanation for the severe symptoms in the non-LVO group is the anatomical location of the infarcts, which included watershed infarcts, pontine perforator infarcts, thalamoperforator infarcts, and bilateral thalamic infarcts. Further, reports have indicated that NIHSS scores can predict the presence of LVO among patients with ischemic stroke arriving at an early time window (within the first hours) but predicts less in late presenting patients (32). The latter was the case for this study and given the fact that our study did not use angiographic techniques to confirm the diagnosis of LVO; this could be another possible explanation for this discrepancy. Nonetheless, the severity of stroke also has multifactorial explanations that include lack of stroke readiness in the healthcare infrastructure and referral networks and lack of community awareness regarding stroke symptoms and signs translating into delays and progression of stroke syndromes. In our cohort, the time from stroke onset to presentation was suboptimal in both groups, with a mean of 1.87 days in the presumed LVO group vs. 1.83 days in the non-LVO group, *p* < 0.32 and no patients arriving within the first 8 h. This delay represents a critical barrier to adopting endovascular interventions and IV thrombolysis in our setting. Therefore, it is a call for prehospital referral strengthening approaches to facilitate transferring potential strokes to a stroke-capable facility.

Also importantly, there were high rates of hemorrhagic transformation on CT head statistically seen more in participants with presumed LVO (46.7% vs. 5.2%, *p* < 0.0001, respectively), even though most of these patients were not parenchymal hematomas (HI 1 and 2). This high rate is multifactorial and could represent the severity of reperfusion injury and uncontrolled hypertension; this highlights the need for specialized stroke units for peri-procedural management of these high-risk patients regardless of whether they undergo mechanical thrombectomy. This also highlights the need for a multidisciplinary approach with the involvement of neurosurgeons to facilitate decompressive hemicraniectomy to manage malignant cerebral edema and hemorrhagic transformation (33).

Finally, there was a higher rate of poor outcomes at 1 year (defined as mRS 4–6) in the presumed LVO group (85%) when compared with the non-LVO group (75.3%; *p* = 0.12), which is concerning but representative of global trends (1, 2). Similarly, 1-year mortality was higher in the presumed LVO group at 80% when compared with the non-LVO group at 73.1%. The overall high rate of poor outcomes in both groups represents a more significant problem with regard to post-stroke care and lack of rehabilitation in LMIC, particularly in SSA. Studies in SSA have previously demonstrated a low implementation of secondary preventive measures following stroke and limited access to rehabilitation services impacting the overall quality of life of these patients (34, 35).

Our study had the following strengths: it provides actionable information given the lack of similar data regarding the burden of presumed LVO in local and regional stroke demographics in LMIC in general and Tanzania as a representative country in SSA in particular. With the development of the MAMC, a state-of-the-art academic hospital sub-serving East and Central Africa, there is potential for more acute therapies for ischemic stroke reperfusion to be introduced into the local neurological landscape. Therefore, the results of this study show important challenges that need to be overcome, as highlighted above. Our study compared presumed large vessel and non-large vessel strokes enabling a unique analysis between these two entities in SSA, while previous data have only looked at overall ischemic strokes.

Finally, the high inter-rater Kappa boosts confidence in the reproducibility of these findings. Given previous infrastructural unavailability of CT/MR angiographic techniques to diagnose and manage LVOs, this study uses the next best method to provide preliminary data that will be the necessary stepping stone to generate discussions among different stakeholders to establish the mechanisms for introducing non-invasive and invasive angiography to diagnose and treat acute ischemic strokes.

This study is limited by the fact that the diagnosis of LVO was made without the use of angiographic techniques, which is the gold standard for this diagnosis. There are reliable data to suggest that the diagnosis of LVO can be inferred from non-angiographic CT studies based on contiguous hypo-density that can be attributed to the involved vascular territory and a compatible clinical syndrome (15, 16). In addition, given later times to presentation/imaging in our cohort, the reliability of NCCT findings increases with time (36, 37). However, it is entirely possible that there were patients in the non-LVO group that had not yet developed contiguous lobar hypodensities due to slow infarct progression; this technique, therefore, has the potential to underdiagnose LVO. Despite the late presentation of our patients (time from the onset of stroke symptoms to hospital arrival of (1.74 days), excluding the 35 patients with normal NCCT head might have underestimated the proportion of presumed LVO. Additionally, our study did not record specific neurological characteristics of these patients.

## Conclusion

There is a high burden of presumed LVO associated with high rates of 1-year morbidity and mortality. Concerted efforts are required to promote cost-effective preventive strategies to combat the looming epidemic of non-communicable diseases and a call for adopting endovascular interventions to reduce morbidity and mortality.

## Data Availability Statement

The raw data supporting the conclusions of this article will be made available by the authors, without undue reservation.

## Ethics Statement

The studies involving human participants were reviewed and approved by Muhimbili University of Health and Allied Sciences Institutional Review Board approval number DA.287/298/01A/. The patients/participants provided their written informed consent to participate in this study.

## Author Contributions

SM, RA, FL, MM, and FS conceptualized and designed the study and drafted the initial manuscript. MC carried out the data analysis and interpreted the results. SM, RA, MC, MM, FS, PM, KK, KT, FL, GR, VG, and AM critically reviewed and revised the final manuscript. All authors contributed to the article and approved the submitted version.

## Funding

This study was funded by the Catholic University of Health and Allied Sciences (Tanzania) and Texas Tech University Health Sciences Center, El Paso TX (USA). The funder has no role in the design, analysis, final write-up of the manuscript, and decision to submit the paper for publication.

## Author Disclaimer

The content is solely the responsibility of the authors and does not necessarily represent the official views of the National Institutes of Health.

## Conflict of Interest

The authors declare that the research was conducted in the absence of any commercial or financial relationships that could be construed as a potential conflict of interest.

## Publisher's Note

All claims expressed in this article are solely those of the authors and do not necessarily represent those of their affiliated organizations, or those of the publisher, the editors and the reviewers. Any product that may be evaluated in this article, or claim that may be made by its manufacturer, is not guaranteed or endorsed by the publisher.

## Abbreviations

AF: Atrial Fibrillation
DALYS: Disability Adjusted Life Years
DWI: Diffusion Weighted Imaging
FBG: Fasting Blood Glucose
HIV: Human Deficiency Virus
LVO: Large vessel occlusion
mRS: Modified Rankin Scale
NIHSS: National Institute of Health Stroke Scale
SSA: sub-Saharan Africa
WHO: World Health Organization.
